# Differences by Sex in Association of Mental Health With Video Gaming or Other Nonacademic Computer Use Among US Adolescents

**DOI:** 10.5888/pcd14.170151

**Published:** 2017-11-22

**Authors:** Hogan H. Lee, Jung Hye Sung, Ji-Young Lee, Jae Eun Lee

**Affiliations:** 1 Madison Central High School, Madison, Mississippi; 2 Jackson State University, Jackson, Mississippi; 3 University of Miami Miller School of Medicine, Miami, Florida

## Abstract

**Introduction:**

Although numerous studies have examined the association between playing video games and cognitive skills, aggression, and depression, few studies have examined how these associations differ by sex. The objective of our study was to determine differences by sex in association between video gaming or other nonacademic computer use and depressive symptoms, suicidal behavior, and being bullied among adolescents in the United States.

**Methods:**

We used data from the 2015 Youth Risk Behavior Survey on 15,624 US high school students. Rao–Scott χ^2^ tests, which were adjusted for the complex sampling design, were conducted to assess differences by sex in the association of mental health with video gaming or other nonacademic computer use.

**Results:**

Approximately one-fifth (19.4%) of adolescents spent 5 or more hours daily on video gaming or other nonacademic computer use, and 17.9% did not spend any time in those activities. A greater percentage of female adolescents than male adolescents reported spending no time (22.1% and 14.0%, respectively) or 5 hours or more (21.3% and 17.5%, respectively) in gaming and other nonacademic computer use (*P* < .001). The association between mental problems and video gaming or other nonacademic computer use differed by sex. Among female adolescents, prevalence of mental problems increased steadily in association with increased time spent, whereas the pattern for male adolescents followed a J-shaped curve, decreasing initially, increasing slowly, and then increasing rapidly beginning at 4 hours or more.

**Conclusion:**

Female adolescents were more likely to have all 3 mental health problems than male adolescents were. Spending no time or 5 hours or more daily on video gaming or other nonacademic computer use was associated with increased mental problems among both sexes. As suggested by the J-shaped relationship, 1 hour or less spent on video gaming or other nonacademic computer use may reduce depressive symptoms, suicidal behavior, and being bullied compared with no use or excessive use.

## Introduction

According to the Entertainment Software Association, in 2016, 63% of American households had at least one person who played video games regularly for 3 or more hours per week, and 27% of players were aged 18 years or younger (1). The average number of hours spent playing games continues to increase. According to Nielsen, time spent playing video games increased from 5.1 hours per week per person in 2011 to 6.3 hours in 2013 (2). 

Internet use among adolescents has increased exponentially in the last decade (3). According to Common Sense Media, in 2015, American teenagers aged 13 to 18 spent an average of 3.5 hours per day on the Internet playing mobile games, watching online videos, using social network sites, chatting, and browsing websites. Moreover, 67% of teenagers owned a smartphone in 2015 (4). Growing ownership of smartphones has influenced the increase in Internet use over time.

Many studies showed that playing video or computer games and using the Internet for nonacademic purposes are associated with social behavior and have examined related health implications for adolescents; however, study results were contradictory. Some studies found that playing games was helpful in improving personality and networks of academic friendships, improving mood, and decreasing stress (5–11). Meta-analytic reviews found that playing violent video games was linked to aggressive behavior and decreased empathy (12–14). Playing violent video games was significantly associated with numerous symptoms of depression among pre-adolescents (15,16). Internet addiction among adolescents, including addiction to social network sites, was also related to sadness, suicide, distress, functional impairment, and cyberbullying (3,17–19).

Researchers and health professionals are concerned about depression, suicide and suicidal behavior, and bullying among children and adolescents (20–22). Being bullied is related to depression, mental illness, violent and aggressive behavior, and suicidal ideation (23–25). Adolescent depression and other mental disorders are chronic health conditions that can continue into adulthood (26). Depression is associated with suicide, and suicide among people aged 15 to 24 years was the third leading cause of death in United States in 2015 at a rate of 12.5 per 100,000 (27).

Although numerous studies have assessed the association between playing video games or other nonacademic computer use and aggression and depression, few studies have examined differences by sex in the relationship between playing video games or other nonacademic computer use and mental health among children and adolescents. Thus, the purpose of this study was to determine how the association between playing video or computer games or other nonacademic computer use (watching online videos, using social network sites, chatting, and browsing websites) and mental health (depressive symptoms, suicidal behavior, being bullied at school or cyberbullied) differs by sex among US adolescents.

## Methods

We used data on 15,624 adolescents from the 2015 Youth Risk Behavior Survey (YRBS), administered by the Centers for Disease Control and Prevention. YRBS, which has been conducted biennially since 1991, uses a 3-stage cluster-sampling design to monitor priority health-risk behaviors among nationally representative samples of private school and public school students in grades 9 through 12 in the United States. In 2015, the sample size was 15,624, the school response rate was 69%, the student response rate was 81%, and the overall response rate was 60%.

Depressive symptoms were defined as the presence of feelings of sadness or hopelessness in response to the question, “During the past 12 months, did you ever feel so sad or hopeless almost every day for two weeks or more in a row that you stopped doing some usual activities?”

Students were questioned on 2 types of bullying: school bullying and cyberbullying. The school bullying question was “During the past 12 months, have you ever been bullied on school property?” with a yes/no answer option. The cyberbullying question was “During the past 12 months, have you ever been electronically bullied? (Include being bullied through e-mail, chat rooms, instant messaging, Web sites, or texting),” also with a yes/no answer option. Being bullied was defined as either being bullied at school or being cyberbullied.

Students were also asked 3 questions related to suicide: had they considered suicide, made a suicide plan, or attempted suicide. The question about considering suicide was “During the past 12 months, did you ever seriously consider attempting suicide?” with a yes/no answer option. The question about making a suicide plan was “During the past 12 months, did you make a plan about how you would attempt suicide?” also with a yes/no answer option. The question about attempting suicide was “During the past 12 months, how many times did you actually attempt suicide?” with response category options of 0 times, 1 time, 2 or 3 times, 4 or 5 times, or 6 or more times. Suicidal behavior was defined as answering yes to the questions about considering suicide or making a suicide plan or if the respondent reported having attempted suicide at least once in the past 12 months.

Engaging in video gaming or other nonacademic computer use was assessed with the question, “On an average school day, how many hours do you play video or computer games or use a computer for something that is not school work? (Count time spent on things such as Xbox, PlayStation, an iPod, an iPad or other tablet, a smartphone, YouTube, Facebook or other social networking tools, and the Internet).” Response options were none, 1 hour or less, 1 hour, 2 hours, 3 hours, 4 hours, and 5 or more hours.

Adjusted and weighted prevalence rates were measured by using a weighting factor in the YRBS to provide nationally representative estimates and by using PROC SURVEYFREQ in SAS version 9.4 (SAS Institute, Inc) to account for the complex 3-stage cluster sampling design. A weighting factor in YRBS data adjusted for school and student nonresponse, sex, grade, and race/ethnicity. Rao–Scott χ^2^ tests, which were adjusted for the complex sampling design by using PROC SURVERYFREQ, were conducted to assess any differences by sex in time spent on video gaming or other nonacademic computer use, depressive symptoms, suicidal behavior, and being bullied and any differences by sex in the association between time spent on video gaming or other nonacademic computer use with depressive symptoms, suicidal behavior, and being bullied. A 2-sided *P* value of <.05 was considered significant.

## Results

Among the sample of 15,624 adolescents, 51.3% were male and 48.7% were female. One in 5 adolescents spent 5 hours or more per day playing video or computer games or used a computer for something unrelated to school work. Almost one-fifth (17.9%) did not engage in playing videos or computer games or other nonacademic computer use. A greater percentage of female adolescents than male adolescents reported no time or 5 hours or more spent in gaming or other nonacademic computer use (*P* < .001) (Table 1).

**Table 1:** Prevalence of Time Spent in Video Gaming and Other Nonacademic Computer Use^a^ and Mental Problems Among Students (N = 15,624), by Sex, 2015 Youth Risk Behavior Survey

Variable^b^	Total, %	Female, %	Male, %	*P* Value^c^
**Time spent, h**				
None	17.9	22.1	14.0	<.001
<1	13.8	10.9	16.5	
1	10.8	10.4	11.2	
2	15.8	13.8	17.7	
3	13.4	12.8	14.0	
4	8.9	8.7	9.1	
≥5	19.4	21.3	17.5	
**Mental problems**				
Depressive symptoms	29.9	39.8	20.3	<.001
Suicidal behavior	21.7	27.3	16.1	<.001
Being bullied	25.7	32.2	19.6	<.001

a Nonacademic computer use includes playing mobile games, watching online videos, using social network sites, chatting, and browsing websites.

b Values are adjusted and percentages are weighted unless otherwise noted.

c Calculated by using the Rao–Scott χ^2^ test.

A significantly higher prevalence of depressive symptoms, suicidal behavior, and being bullied was observed among female adolescents than male adolescents (Table 1). Approximately 1 in 3 adolescents had depressive symptoms; 1 in 5 had considered suicide, made a suicide plan, or attempted suicide; and 1 in 4 had been bullied at school or had been cyberbullied. The prevalence of depressive symptoms, suicidal behavior, and being bullied differed significantly by sex (*P* < .001 for each mental health problem). Female adolescents were nearly twice as likely to have depressive symptoms, suicidal behavior, and to have been bullied than male adolescents.

A pattern of change in the prevalence of depressive symptoms, suicidal behavior, and being bullied in relation to time spent on video gaming or other nonacademic computer use had a J-shaped curve (Figure). Prevalence decreased initially, then increased slowly, and then increased rapidly from 4 hours or more. Those spending 5 or more hours per day on video games or other nonacademic computer use had the highest prevalence of depressive symptoms (43.1%), suicidal behavior (32.4%), and being bullied (31.5%). The lowest prevalence of depressive symptoms (22.8%) and being bullied (21.9%) was among those spending less than 1 hour, and the lowest prevalence of suicidal behavior was among those spending 1 hour (15.7%).

**Figure:**
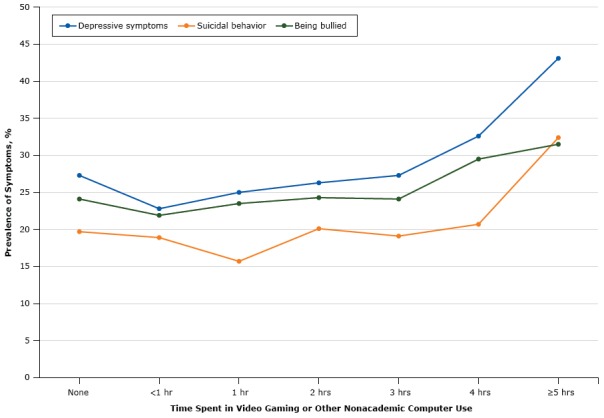
Prevalence of depressive symptoms, suicidal behavior, and being bullied in relation to time spent on video gaming or other nonacademic computer use among male and female adolescents, Youth Risk Behavior Survey, 2015. **Table:** 

Mental Health	Time Spent in Video Gaming or Other Nonacademic Computer Use						
	None	<1 h	1 h	2 h	3 h	4 h	≥5 h
Depressive symptoms, %	27.3	22.8	25.0	26.3	27.3	32.6	43.1
Suicidal behavior, %	19.7	18.9	15.7	20.1	19.1	20.7	32.4
Being bullied, %One	24.1	21.9	23.5	24.3	24.1	29.5	31.5

The relationship between mental health and hours spent in video gaming or other nonacademic computer use varied by sex and type of mental health problems (Table 2). The percentage of female adolescents experiencing depressive symptoms with no time spent in video gaming or other nonacademic computer use was 32.7%, rose to 33.3% for less than 1 hour spent, fell to 32.7% for 1 hour spent, and then rose steadily to a peak of 53.8% at 5 or more hours spent (*P* < .001). Prevalence among male adolescents was 19.0% for no time spent in video gaming or other nonacademic computer use, fell to 16.1% for less than 1 hour, than rose to 30.3% for 5 hours or more (*P* < .001). For suicidal behavior among female adolescents, prevalence was 22.5% at no hours spent and rose to 37.8% at 5 or more hours spent. For male adolescents, prevalence was 14.9% for no hours spent and rose to 25.1% for 5 or more hours spent. Female adolescents who spent no time in video gaming or other nonacademic computer use had a prevalence of 27.3% of being bullied and 36.8% at 5 or more hours spent. For male adolescents, the prevalence of being bullied was 19.0% at no time spent, fell to 15.3% for less than 1 hour spent, and then rose to 25.5% for 5 or more hours spent. For all mental health problems and both sexes, prevalence fluctuated up and down between less than 1 hour and 5 or more hours and generally increased beginning at 4 hours.

**Table 2:** Prevalence of Mental Problems by Time Spent in Video Gaming and Other Nonacademic Computer Use^a^ Among Students (N = 15,624), by Sex, 2015 Youth Risk Behavior Survey

Variable	No. of Hours Spent, %^b^							*P *Value^c^
	None	<1	1	2	3	4	≥5	
**Depressive symptoms**								
Female	32.7	33.3	32.7	36.1	38.4	45.9	53.8	<.001
Male	19.0	16.1	18.1	18.9	17.3	20.6	30.3	
**Suicidal behavior**								
Female	22.5	25.9	19.5	26.1	25.4	28.5	37.8	<.001
Male	14.9	14.3	12.5	15.3	13.5	13.7	25.1	
**Being bullied**								
Female	27.3	32.2	30.4	33.0	29.3	37.4	36.8	<.001
Male	19.0	15.3	17.3	17.7	19.6	22.3	25.5	

a Nonacademic computer use includes playing mobile games, watching online videos, using social network sites, chatting, and browsing websites.

b Values are adjusted and percentages are weighted unless otherwise noted.

c Calculated by using the Rao–Scott χ^2^ test.

## Discussion

Our study examined differences between male and female adolescents in time spent video gaming or other nonacademic computer use, depressive symptoms, being bullied, and suicidal behavior and any differences by sex in the association of time spent in video gaming or other nonacademic computer use with depressive symptoms, being bullied, and suicidal behavior. Our study provided evidence of the J-shaped relationship between video gaming or other nonacademic computer use and depressive symptoms, suicidal behavior, and being bullied among US adolescents. Adolescents who spent 5 hours or more in video gaming or other nonacademic computer use had the highest rates of depressive symptoms, suicidal behavior, and being bullied. The lowest rates were among adolescents spending less than 1 hour or 1 hour. Adolescents who did not play video or computer games or use the computer for nonacademic reasons had higher rates than those who spent 1 hour or less per day. However, female adolescents were almost twice as likely to experience depressive symptoms, suicidal behavior, or being bullied in relation to time spent playing video games or in other nonacademic computer use than male adolescents.

One study found similar results about the relationship between video gaming or other nonacademic computer use and suicide by using the 2007 and 2009 YRBS (19). That study found that 5 hours or more of daily video gaming or other nonacademic computer use was associated with higher risk of sadness, suicidal ideation, and suicidal planning than no time spent. The same study also found that 1 hour or less of daily video gaming had potentially protected against 2-week sadness compared with no video gaming. However, that study did not investigate differences by sex in the associations. It also did not investigate the association between being bullied and daily video gaming or other nonacademic computer use. Because Internet technologies have developed rapidly, adolescents are able to easily acquire information, and they have many ways, such as social network sites, to communicate with others online, which may suggest that adolescents are more likely to be at risk of being bullied, especially of being cyberbullied. Our study consistently showed that adolescents who spent 4 hours or more daily on video games or other nonacademic computer use were 1.5 times more likely to be bullied than those who spent 3 hours or less.

Two studies, Belanger et al and Kim, found a U-shaped association between Internet use for nonacademic purposes and mental health among Swiss and Korean adolescents, respectively (28,29). Both studies suggested that health professionals should be alert to heavy Internet use (≥2 h/d) and to no use as indicators of high risk for mental disorders. However, both studies defined heavy use as spending 2 hours or more daily on the Internet. Because Belanger et al used data from 2002 and Kim used data from 2009, their categories for intensity of Internet use are not relevant to recent trends, which were reported in 2015 at 3.5 hours per day on average for US adolescents, including playing mobile games, watching online videos, using social network sites, chatting, and browsing websites (4).

Although YRBS data have the advantage of being a nationally representative sample of adolescents, our study has several limitations. First, because YRBS consists of cross-sectional data, assessing the cause–effect relationship between video gaming or other nonacademic computer use and mental problems was not possible. Second, investigating the association of mental problems with video gaming or other nonacademic computer use separately was not possible. Although video gaming and other nonacademic computer use are different measures, YRBS uses a single variable for the 2 activities. However, studies have demonstrated differences between the 2 measures. For example, in one study, 62% of male adolescents enjoyed playing video games, compared with 20% of female adolescents, and 44% of female adolescents enjoyed using social media, compared with 29% of male adolescents (4). Moreover, on average, female adolescents spent about 40 minutes more on social network sites than male adolescents (4). Further research is warranted for establishing a separate measure each for video gaming and other nonacademic computer use to determine their relation to mental health.

Our study found that video gaming or other nonacademic computer use among US adolescents for 5 hours or more daily was significantly associated with increases in depressive symptoms, suicidal behavior, and being bullied. The prevalence of each of the 3 mental health problems was higher among female adolescents than among male adolescents. As suggested by the J-shaped relationship, 1 hour or less of playing video games or other nonacademic computer use may reduce the prevalence of these mental health problems whereas nonuse or excessive use may increase them. Therefore, sex-specific intervention programs should be developed. Furthermore, because our data show that some video gaming and other nonacademic computer use may reduce the prevalence of depressive symptoms, suicidal behavior, and being bullied, public health professionals may want to shift mindfulness intervention programs toward eHealth or mHealth technologies rather than completely dismissing the activities. Use of technology for health promotion and disease prevention has advanced rapidly through the emergence of eHealth and mHealth technologies. Both technologies offer several advantages over traditional, in-person methods of health promotion and disease prevention interventions. Both are cost efficient and interactive and can automate delivery of interventions, thereby enabling real-time assessments, personalizing and tailoring content, and reaching larger populations and hard-to-reach subgroups than conventional methods (30). Sex-specific mindfulness intervention programs that use these technologies in conjunction with video games and other nonacademic computer use may be well received by adolescents as well as by their parents and teachers.
